# Supplementary material for: Splice-Junction-Based Mapping of Alternative Isoforms in the Human Proteome
Source: Cell Rep. Author manuscript; Available in PMC 2020 Jan 15. (PMC6961840; doi:10.1016/j.celrep.2019.11.026)
Supplement: 2 [file NIHMS1546469-supplement-2.zip › DF1/PXD006675/LeftCoronaryArtery/LeftCoronaryArtery_8_CAPN7_KVYYPGIFSMQLYFFK.pdf]

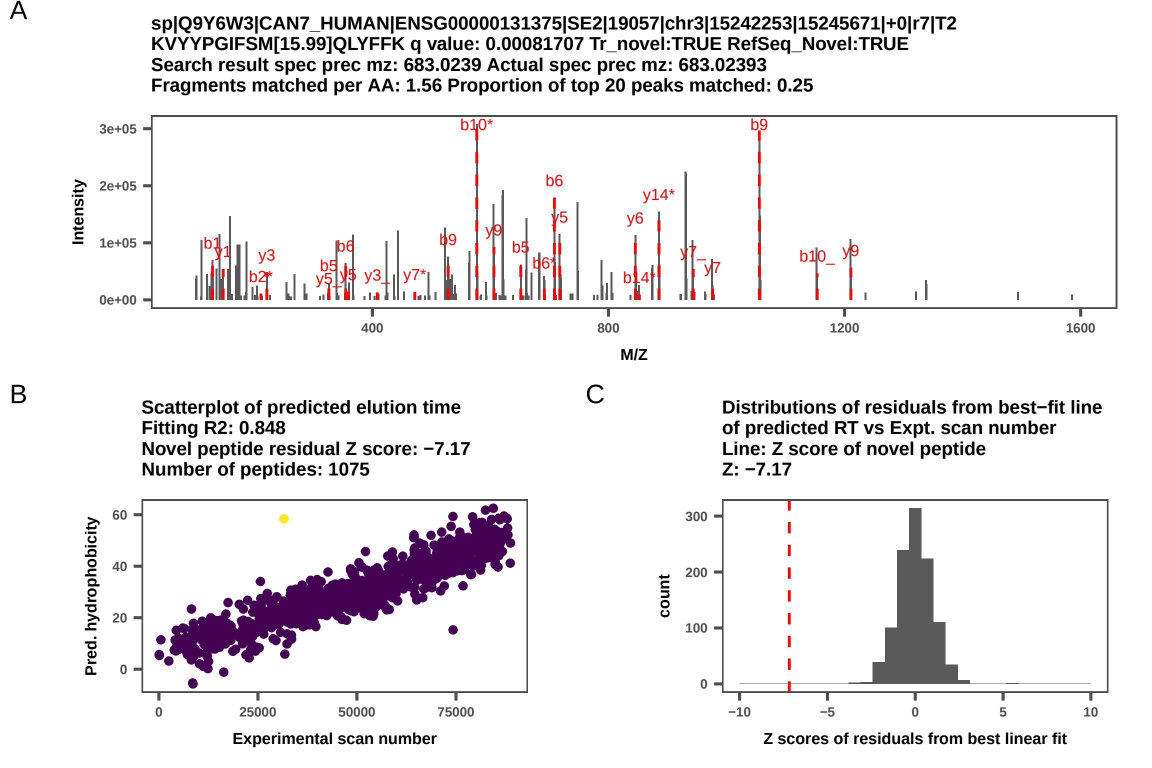
A
sp|Q9Y6W3|CAN7_HUMAN|ENSG00000131375|SE2|19057|chr3|15242253|15245671|+0|r7|T2
KVYYPGIFSM[15.99]QLYFFK q value: 0.00081707 Tr_novel:TRUE RefSeq_Novel:TRUE
Search result spec prec mz: 683.0239 Actual spec prec mz: 683.02393
Fragments matched per AA: 1.56 Proportion of top 20 peaks matched: 0.25
Intensity
3e+05
2e+05
1e+05
0e+00
b10*
b9
b6
y14*
y5
y6
y9
b9
b5
b6*
y7_
y7
b10_
y9
b1
y1
y3
b2*
b5
y5_
y5
b6
y3_
y7*
b14*
400
800
1200
1600
M/Z
B
Scatterplot of predicted elution time
Fitting R2: 0.848
Novel peptide residual Z score: −7.17
Number of peptides: 1075
Pred. hydrophobicity
60
40
20
0
0
25000
50000
75000
Experimental scan number
C
Distributions of residuals from best−fit line
of predicted RT vs Expt. scan number
Line: Z score of novel peptide
Z: −7.17
count
300
200
100
0
−10
−5
0
5
10
Z scores of residuals from best linear fit
